# CSE1L, as a novel prognostic marker, promotes pancreatic cancer proliferation by regulating the AKT/mTOR signaling pathway

**DOI:** 10.7150/jca.54482

**Published:** 2021-03-14

**Authors:** Xiao Zhang, Xiaofei Zhang, Tiebo Mao, Haiyan Xu, Jiujie Cui, Hechun Lin, Liwei Wang

**Affiliations:** State Key Laboratory of Oncogenes and Related Genes, Shanghai Cancer Institute, Department of Oncology, Renji Hospital, School of Medicine, Shanghai Jiao Tong University, Shanghai, China.

**Keywords:** pancreatic cancer, CSE1L, proliferation, AKT/mTOR signaling pathway.

## Abstract

Pancreatic cancer is one of the most aggressive tumors with poor prognosis and new targetable therapies are urgently required. CSE1L (chromosome segregation 1 like) is thought to play an important role in tumorigenesis and acts as a cancer therapeutic target. However, the biological function and the underlying mechanism of CSE1L in pancreatic cancer are still not fully explicit. In the present study, we found that high CSE1L expression was related to a worse prognosis in patients with pancreatic cancer according to data from the Cancer Genome Atlas (TCGA) database. Additionally, we found that CSE1L knockdown inhibited the proliferation of pancreatic cancer cells and promoted apoptosis, while CSE1L overexpression demonstrated the opposite phenomenon. Furthermore, we discovered that CSE1L might regulate pancreatic cancer proliferation through AKT signaling pathway. In summary, we reveal that CSE1L plays a crucial role in tumor growth and may serve as a potential prognostic and therapeutic target for pancreatic cancer.

## Introduction

Pancreatic cancer is the leading cause of cancer-related deaths worldwide. Of the new cancer cases that occurred worldwide in 2018, pancreatic cancer was the seventh leading cause of cancer-related death in both males and females with almost as many deaths as cases 1. Besides, it is expected to become the second leading cause of cancer-related mortality in the United States by 2030 2. According to existing treatments for patients suffering from pancreatic cancer, the 5-year survival rate remains remarkably low, at approximately 9% 1, 3. However, the complex molecular and cellular mechanisms involved in pancreatic cancer have not been fully elucidated. Therefore, it is urgent to explore the mechanism and clarify new targets for pancreatic cancer treatment.

Malfunction of nuclear-cytoplasmic transport is deemed to contribute to tumorigenesis mediated by proteins among which exportins exhibit tremendous function. Exportins include the following seven members: XPO1, CSE1L (XPO2), XPOT, XPO4, XPO5, XPO6 and XPO7 4. Overexpression of exportins has been reported among a range of cancers, with impacts on cell growth, differentiation, drug resistance, and tumor microenvironment 4, 5. However, the involvement of the exportins in pancreatic cancer remains unknown, except for XPO1. Here, we analyzed the mRNA expression levels of these exportins using the GEO, UALCAN and ONCOMINE databases. According to the above databases, CSE1L was the only exportin that overexpressed in pancreatic cancer tissues. Therefore, we identified CSE1L as the exportin with the greatest potential that may play vital roles in pancreatic cancer.

CSE1L (chromosome segregation 1 like), also known as CAS, CSE1 or XPO2, is an exportin. The CSE1L gene, located on chromosome 20q13, encodes a protein of 100-kDa molecular weight 6. CSE1L functions as an oncogene in certain types of tumors, including hepatocellular carcinoma 7, gastric cancer 8, colorectal cancer 9, breast cancer 10 and ovarian cancer 11, oral cancer 12 and osteosarcoma 13. However, the role and underlying mechanism of CSE1L in pancreatic cancer is still not fully explicit.

In the present study, we examined CSE1L expression in pancreatic cancer tissues using three online datasets and our own tissue samples. We also analyzed prognosis of CSE1L expression and its correlation with the clinicopathological features of pancreatic cancer. Next, we performed loss-of-function and gain-of-function assays to explore the function of CSE1L of pancreatic cancer in vitro and *in vivo*. We demonstrated that CSE1L promoted the proliferation of pancreatic cancer. In addition, we revealed that CSE1L participates in the AKT signaling pathway.

Taken together, our findings suggest that CSE1L offers significant clues for further study of the mechanism and clinical therapy of pancreatic cancer.

## Materials and methods

### Clinical samples and immunohistochemical staining (IHC)

90 pancreatic cancer tissue samples and 79 corresponding non-tumor tissue samples were collected from the Department of Pathology at the First Affiliated Hospital of Nanjing Medical University, between January 2012 and December 2016. This study was approved by the Research Ethics Committee of Renji Hospital, School of Medicine, Shanghai Jiao Tong University and the First Affiliated Hospital with Nanjing Medical University. Written informed consent was obtained from all participants. This study was performed in accordance with the principles of the Declaration of Helsinki. All of the patients were diagnosed, and the diagnosis was confirmed by histology. Tissue microarrays was constructed by Shanghai Outdo Biotech Company (China). Clinicopathological information included age, gender, clinical stage, invasion depth, lymph node metastasis, distant metastasis and tumor differentiation. The median age was 62 years (range: 31-78 years).

Tissue microarray were deparaffinized in xylene and rehydrated in a graded alcohol. Then, the sections were blocked with 3% H_2_O_2_, and antigen retrieval was performed by heating the sections in citrate buffer. The sections were incubated with an antibody against CSE1L (Proteintech) at 4°C overnight, followed by incubation with biotinylated secondary antibodies. Assessment of IHC staining was independently performed by two expert pathologists. CSE1L protein expressions were scored according to staining intensity and the percentage of positive cells. The percentage of positive cells was scored as follows: 0% (0), 1-10% (1), 11-50% (2), 51-80% (3), 81-100% (4). Staining intensity was scored as follows: no staining (0), week (1), moderate (2), and strong (3). Comprehensive score = staining percentage × intensity. CSE1Lprotein expression was classified as follows: < 6 low expression, ≥ 6 high expression.

### Cell lines and cell culture

Five cell lines were used, Human normal pancreatic ductal cells HPNE and pancreatic cancer cell lines PANC1, AsPC1, CFPAC1 and human embryonic kidney cells 293T. HPNE and Human embryonic kidney 293T cells were obtained from the American Type Culture Collection (ATCC). PANC1, AsPC1, and CFPAC1 were obtained from the Cellular Institute of Chinese Academy of Science (Shanghai, China). These cells were incubated in medium supplemented with 10% FBS (Biowest, South America), 100 µ/ml penicillin (Sigma-Aldrich), and 100 µg/ml streptomycin (Sigma-Aldrich). DMEM, RPMI 1640 and IMDM were used for PANC1, AsPC1, and CFPAC1, respectively. All cells were incubated at 37°C in humidified air with 5% CO_2_.

### RNA isolation and quantitative real-time PCR assays

Total RNA samples from the pancreatic cell lines used in this study were extracted with TRIzol reagent (Invitrogen, CA) according to the manufacturer's protocol and were quantified with Nanodrop 2000 (Thermo, Japan). A PrimeScript RT Reagent Kit (TaKaRa, China) was used to synthesize first-strand cDNA, which was used as the template for real-time polymerase chain reaction (qPCR). qPCR was performed three times with SYBR Green Premix Ex Taq (TaKaRa, China). The specific primers for the target genes are listed in Supplementary Table S1. β-actin was used as the internal control.

### RNA interference

siRNA oligonucleotides targeting CSE1L were designed and synthesized by RiboBio (Guangzhou, China). The sequences of the siRNAs used were as follows: si-CSE1L-1, GGATAATGTTATCAAAGTA; si-CSE1L-2, CGACGGTATCAAATATATT. Scrambled siRNAs targeting no known gene sequences were used as the negative control. Lipofectamine 3000 Reagent (Invitrogen) was used to conduct siRNA transfection according to the manufacturer's protocol. After transfection for 48 h, the cells were used for RNA extraction, immunoblotting and viability assays.

### Plasmid construction and transfection

Full-length CSE1L cDNA was cloned and inserted into the lentiviral expression vector pWPXL (Addgene). To produce the intended lentivirus, the constructed plasmids and lentiviral vector packaging system were transfected into HEK-293T cells using Lipofectamine 2000. After 48 hours, the supernatants from the HEK-293T cultures were collected to infect PANC1 cells/AsPC1 cells.

### In vitro cell proliferation assays

For the proliferation assay, 48 h after siRNA transfection, cells were diluted with complete medium and then, 5000 cells were seeded into each well of a 96-well plate and incubated. A 10 μL aliquot of Cell Counting Kit-8 (CCK8) (Dojindo) was added to triplicate wells, and incubated for 1-2 h, and absorbance was measured at 450 nm. Each measurement was performed in triplicate, and experiments were repeated twice.

For the colony formation assay, 48 hours after siRNA transfection, PANC1 or AsPC1 or CFPAC1 cells (2000 cells/well) were seeded in 6-well plates. After 10-14 days of incubation, cells in each well were fixed with 100% methanol for 20 min, and stained with 0.1% crystal violet for 20 min, and cell colonies were counted. All assays were independently performed in triplicate.

### Apoptosis assays

Forty-eight hours after siRNA transfection, cells and medium were collected. Cells were stained with Annexin V-FITC/AAD and propidium iodide (BD Biosciences) in the dark at room temperature. After 15 min, the cells were analyzed using a FACSCalibur flow cytometer (BD Biosciences). The results were analyzed using FlowJo software. Assays were independently performed three times.

### Western blot analysis

The lysates were extracted from cultured cells and then separated using 8% or 10% sodium dodecyl sulfate-polyacrylamide gel electrophoresis (SDS-PAGE), followed by transferring onto nitrocellulose membranes (Millipore). Next, we blocked the membranes in 5% milk with phosphate-buffered saline. After one hour of blocking, the membranes were incubated with primary antibodies (Supplementary Table 2) at 4°C overnight. The next day, the membranes were incubated with HRP-conjugated anti-mouse IgG (Sigma-Aldrich) or HRP-conjugated anti-rabbit IgG (Sigma-Aldrich) secondary antibodies. Subsequently, visualization was performed with SuperSignal West Femto Maximum Sensitivity Substrate (Thermo, USA).

### Animal experiments

All methods were performed in accordance with the guidelines for animal experimentation and approved by the Experimental Animal Ethics Committee of Shanghai Jiaotong University. The stably transfected cell lines PANC1-pWPXL and PANC-CSE1L-OE (4×106 cells/each) were subcutaneously implanted into BALB/c nude mice, respectively. A total of 12 nude mice were used with 6 mice in each group. Tumor size was measured every twice a week and calculated using the following equation: Volume (mm^3^) = (length × width 2)/2. After 50 days, the mice were euthanized, and the tumors were excised and weighed.

### Statistical analysis

The data were imaged with GraphPad Prism 5 software. The quantitative variables were presented as the means and S.E.M. Student's *t* test or Chi-square tests were used between the two groups. P< 0.05 was considered statistically significant. All analyses were performed with SPSS software (version 19.0).

## Results

### CSE1L is overexpressed in pancreatic cancer patients

**Figure 1:**
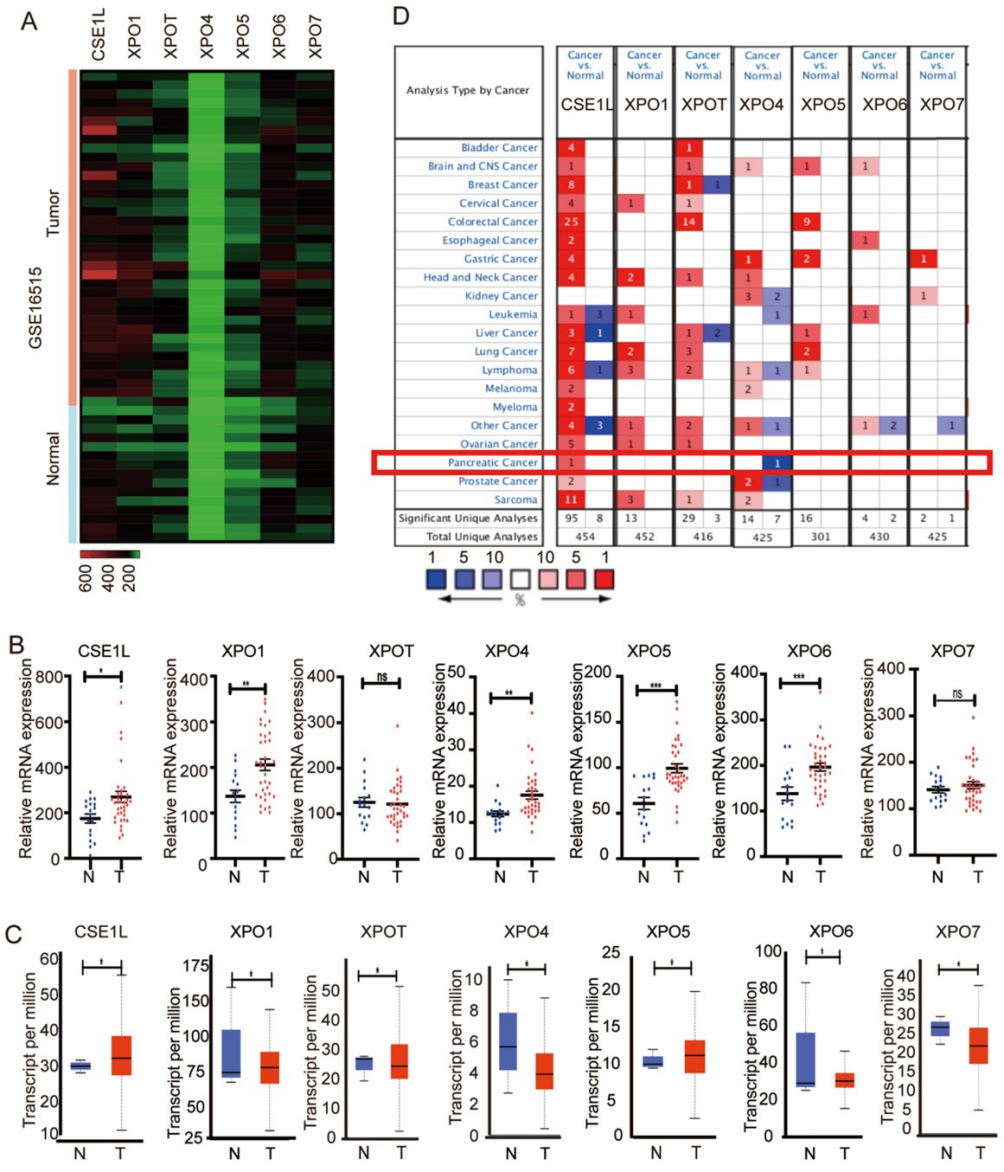
** Aberrant expression level of exportins in pancreatic cancer patients.** (A) Heatmap of the seven exportins mRNA expression in GSE16515. (B) The mRNA expression of seven exportins in pancreatic cancer tissues assessed using data from GSE16515. (C) Expression panels for seven exportins comparing the data between 4 normal individuals and 178 pancreatic cancer patients in the TCGA database. (D) Seven exportins mRNA expression levels in 20 different cancer types by using the ONCOMINE database. *P < 0.05, **P < 0.01, ***P < 0.001.

To explore the expression of the seven exportins in pancreatic cancer, we analyzed their mRNA expression levels using data from GSE16515 (https://www.ncbi.nlm.nih.gov/gds), the Cancer Genome Atlas (TCGA) database (https://portal.gdc.cancer.gov/) and the ONCOMINE database (https://www.oncomine.org/). First, we mined the differentially expressed genes between pancreatic cancer tissues and adjacent normal tissues in the GSE16515 dataset. As is shown in Fig. 1A and B, compared with normal pancreatic tissues, tumor tissues had significant overexpression of CSE1L, XPO1, XPO4, XPO5, and XPO6. Next, the gene expression landscape of XPOs were measured by UALCAN (http://ualcan.path.uab.edu/), resources of which were based upon data from 31 cancer types of the TCGA database. As is shown in Fig. 1C, CSE1L, XPO1, XPO5 and XPO6 exerted higher mRNA expression in pancreatic cancer tissues compared to nontumorous tissues. Moreover, the ONCOMINE database showed that CSE1L exhibited higher mRNA expression in pancreatic cancer tissues (Fig. 1D). According to the above databases, CSE1L was the only exportin that upregulated in pancreatic cancer tissues. Therefore, we identified CSE1L as the research subject.

### Clinical significance of CSE1L in patients with pancreatic cancer

**Figure 2:**
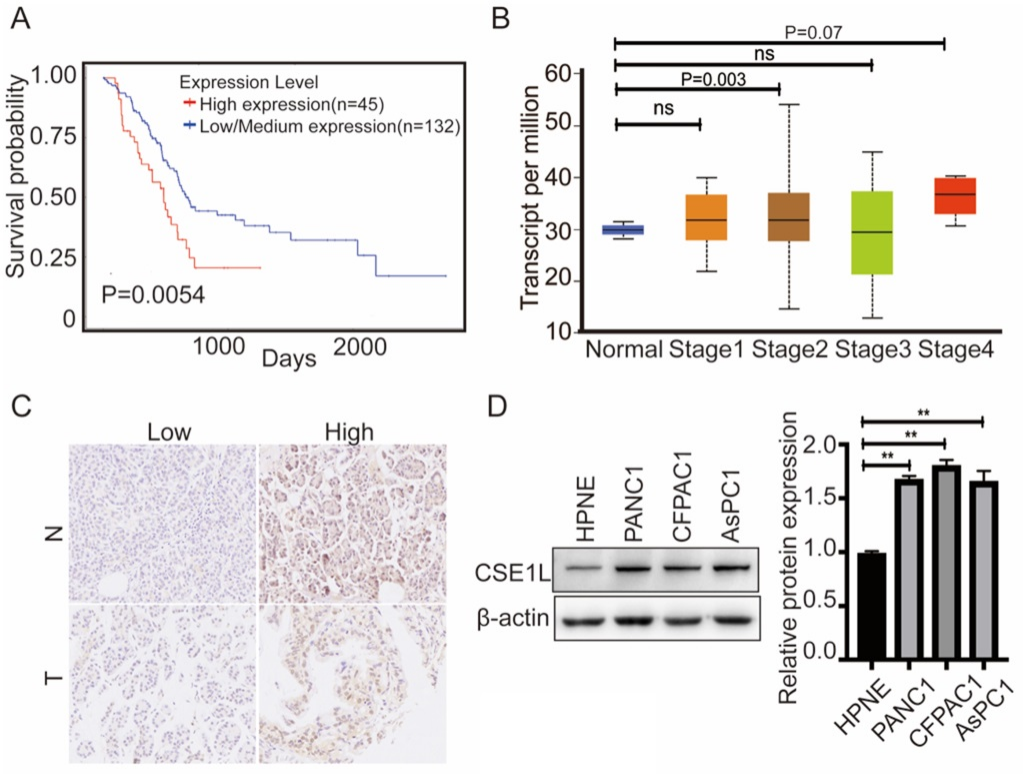
** CSE1L is significantly overexpressed in both pancreatic cancer tissues and cell lines.** (A)The prognostic value of the CSE1L expression level in pancreatic cancer patients; the survival curve was plotted utilizing the UALCAN database. (B) Relationship between the CSE1L expression level and pancreatic cancer clinical stages. (C) CSE1L is overexpressed in pancreatic cancer tissues. (D) CSE1L is overexpressed in pancreatic cancer cell lines.

Furthermore, to better understand the impact of CSE1L gene expression on the overall survival of pancreatic cancer patients, we utilized the TCGA data to assess the prognostic value of CSE1L. The database showed that higher CSE1L expression was associated with worse outcomes in pancreatic cancer patients (*P* = 0.0054) (Fig. 2A).We next analyzed the correlation between CSE1L expression levels and the clinicopathological parameters of pancreatic cancer patients by the UALCAN database. As shown in Fig. 2B, the mRNA expression level of CSE1L in stage II groups were remarkably upregulated compared with that in normal tissues. To further determine the clinicopathological significance of CSE1L in pancreatic cancer, we performed IHC analysis of CSE1L in a tissue microarray that included an independent set of 90 cases of pancreatic cancer tissues. Representative IHC images of CSE1L expression are shown in Fig. 2C. The CSE1L protein was localized in both cell nucleus and cytoplasm. Of the 90 tumor tissue samples, high CSE1L protein levels were found in 60% (54 of 90) of pancreatic cancer tissues, compared with only 35.4% (28 of 79) of normal tissues (p = 0.0013). Correlations between the CSE1L expression level and the clinicopathological characteristics of patients with pancreatic cancer are summarized in Table 1.

### CSE1L promotes pancreatic cancer growth in vitro and *in vivo*

**Figure 3:**
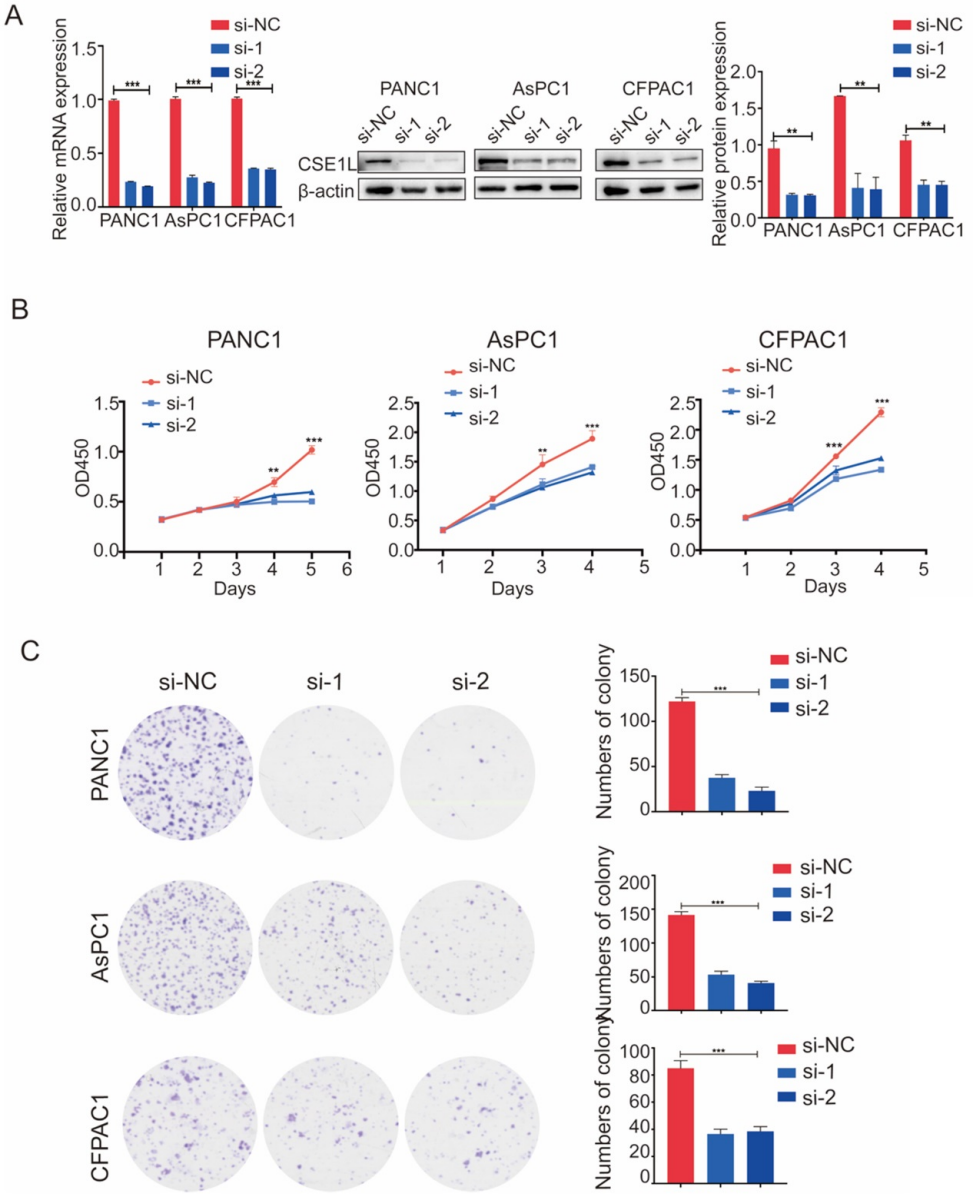
** Knockdown of CSE1L inhibits pancreatic cancer cell growth.** (A) Western blot analyses showing the efficiency of CSE1L knockdown in PANC1 cells and AsPC1 cells, respectively. (B and C) Knockdown of CSE1L significantly suppressed the proliferation of PANC1, AsPC1 and CFPAC1 cells by CCK8 and colony formation assays. Data are representative of results from three independent experiments. *P < 0.05, **P < 0.01, ***P < 0.001.

**Figure 4:**
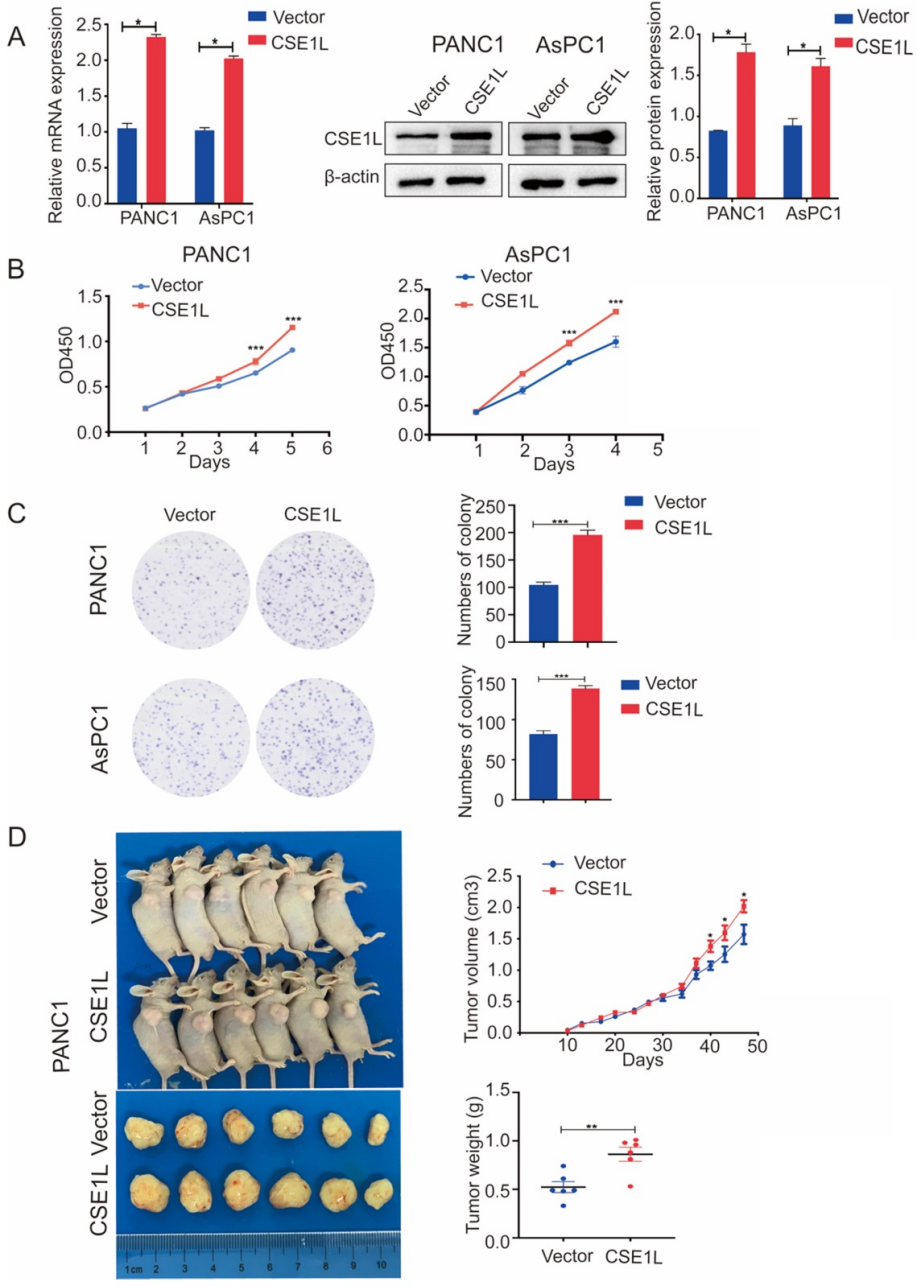
** Overexpression of CSE1L promoted pancreatic cancer growth.** (A)Western blot analyses showing the efficiency of CSE1L overexpression in PANC1 cells and AsPC1 cells, respectively. (B and C) Overexpression of CSE1L significantly promoted proliferation in PANC1 and AsPC1 cells by CCK8 and colony formation assays. Data are representative of results from three independent experiments. (D) CSE1L increases pancreatic cancer growth *in vivo*.*P < 0.05, **P < 0.01, ***P < 0.001.

The expression of CSE1L was much higher in pancreatic cancer cell lines than in normal human pancreatic ductal cells (Fig. 2D). To investigate the functional roles of CSE1L in pancreatic cancer progression, we knocked down CSE1L via independent siRNAs significantly in PANC1, AsPC1, CFPAC1 cells. The knockdown efficiency of CSE1L was validated by qPCR and western blotting assays (Fig. 3A). Next, the results of CCK8 and colony formation assays showed that knockdown of CSE1L significantly suppressed proliferation in PANC1, AsPC1 and CFPAC1 cells (Fig. 3B and C). Conversely, stable cell lines (CSE1L) were established via lentiviral infection in PANC1 and AsPC1 cell lines. The efficiency of CSE1L overexpression was validated by qPCR and western blotting assays (Fig. 4A). Overexpression of CSE1L significantly enhanced the proliferation of PANC1 and AsPC1 cells (Fig. 4 B and C). Moreover, a subcutaneous tumor transplantation test showed that overexpression of CSE1L promoted the proliferation *in vivo* (Fig. 4D). Taken together, these results indicated that CSE1L acted as an important tumor driver in pancreatic cancer proliferation.

### CSE1L inhibits apoptosis in pancreatic cancer cells

**Figure 5:**
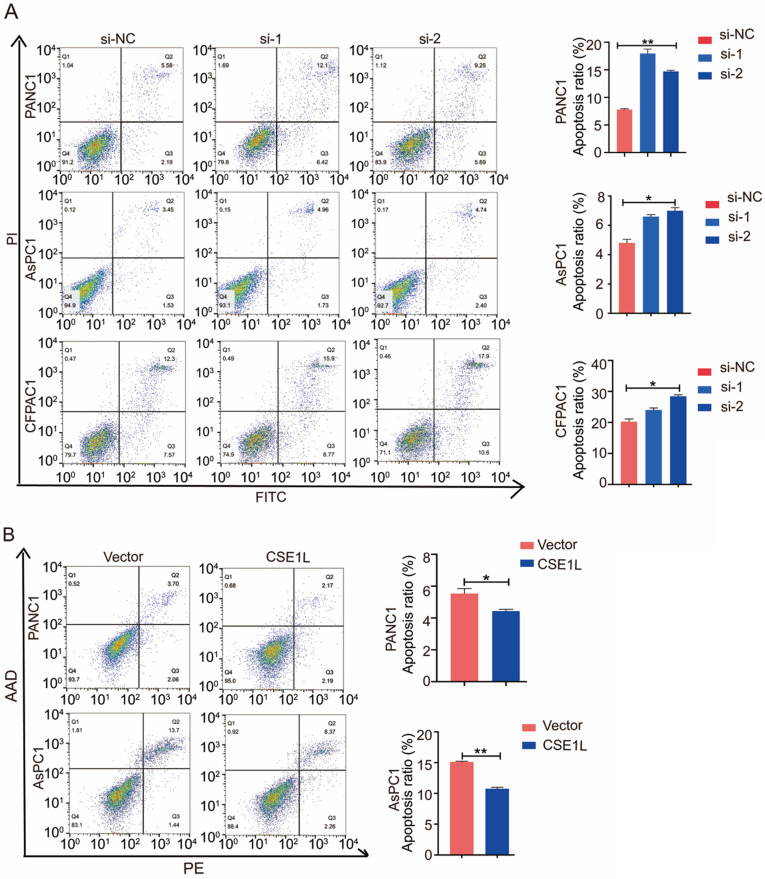
** CSE1L influences apoptosis of pancreatic cancer cells.** (A) CSE1L knockdown in PANC1, AsPC1 and CFPAC1 cells significantly increased the percentage of apoptotic cells. (B) Overexpression of CSE1L significantly decreased the percentage of apoptotic cells. *P < 0.05, **P < 0.01.

CSE1L has been reported to influence apoptosis in several cancers 8. Therefore, we performed the flow cytometry analysis to determine the effect of CSE1L on apoptosis in different pancreatic cancer cells. As shown in Fig 5A and B, CSE1L knockdown in PANC1, AsPC1 and CFPAC1 cells significantly increased the percentage of apoptotic cells. Upon lentiviral overexpression of CSE1L, apoptotic cell death was considerably inhibited.

### CSE1L regulated the AKT/mTOR signaling pathway in pancreatic cancer

**Figure 6:**
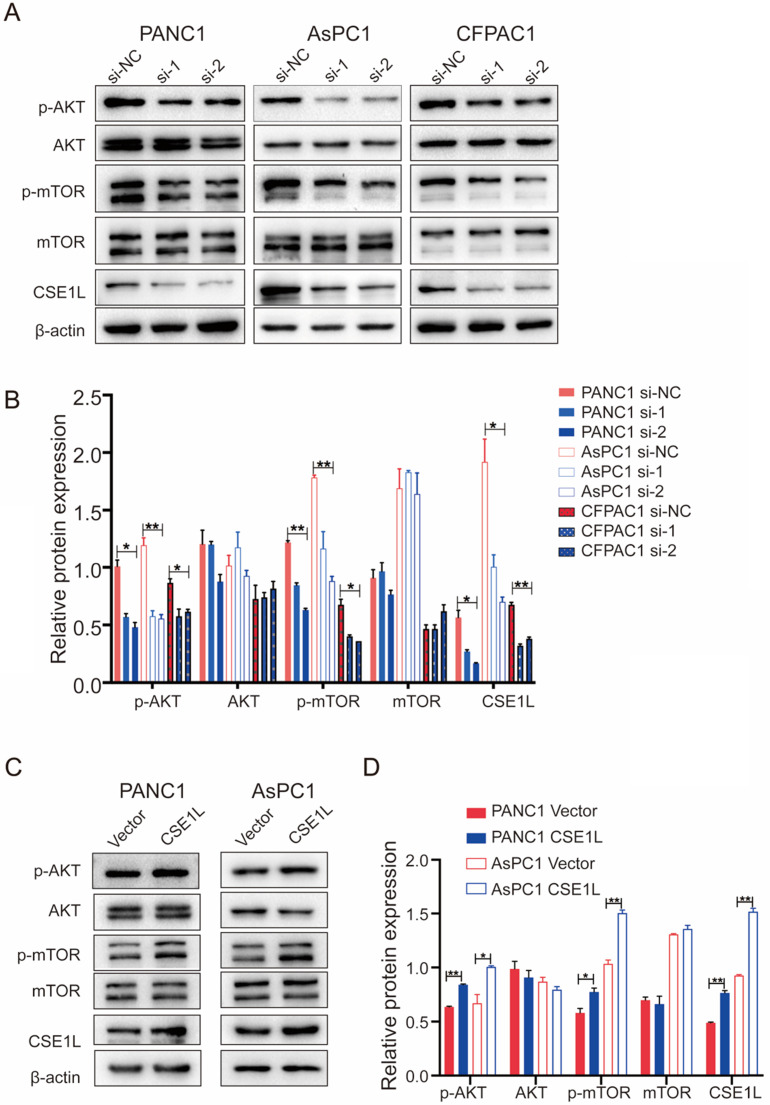
** CSE1L regulates the AKT signaling pathway in pancreatic cancer cells.** (A) Western blot analysis of p-AKT, AKT, p-mTOR and mTOR in cells transfected with CSE1L siRNAs. (B) Western blot analysis of p-AKT, AKT, p-mTOR and mTOR in cells stably over-expressing CSE1L. *P < 0.05, **P < 0.01.

AKT/mTOR signaling is involved in CSE1L-mediated cancer growth 12. Therefore, we detected the effect of CSE1L on the AKT/mTOR signaling pathways through western blotting in pancreatic cancer cell lines. As shown in Fig. 6 A and B, CSE1L knockdown inhibited the phosphorylation of AKT (Ser473) and mTOR (Ser2448) without influencing the total protein levels of both AKT and mTOR, whereas overexpression of CSE1L promoted phosphorylation of the corresponding sites of AKT and mTOR (Fig. 6 C and D). Therefore, these results show that the AKT/mTOR signaling pathway is crucial for CSE1L-mediated pancreatic cancer proliferation.

## Discussion

Pancreatic cancer is an extremely aggressive disease with worse outcomes. Despite a variety of studies exploring its biological mechanisms, the overall survival rate of PDAC patients has remained unchanged in recent years 14-16. Therefore, it is urgent to explore new therapeutic targets for pancreatic cancer.

In recent years, many studies have shown that the expression of exportins is aberrant in numerous human cancers 17. Identification of tumor-associated exportins is critical in tumorigenesis and may serve as novel therapeutic targets. XPO1 dysregulation indirectly regulates cellular functions such as cell proliferation, cellular transformation, apoptosis, and chromosome segregation 18. In addition, XPO1 protein overexpression has been observed in glioma, gastric cancer, ovarian carcinoma, osteosarcoma and other cancers 19. Furthermore, many inhibitors have been developed to block one of the exportins, XPO1. XPO1 inhibitors block nuclear export of XPO1 cargos, such as p53, p27, and eIF4E. Nuclear retention of these regulators resulted in subsequent activation of apoptotic- and stress-related gene expression 5, 20. Besides, Vaidyanathan et al identified Exportin 2/CSE1L, Exportin 3/XPOT, Exportin 5/XPO5, and RANBP1 as novel potential targets of breast cancer by using publicly available datasets 21. Chen et al identified high expression of XPO1, CSE1L, XPOT, XPO4/5/6 was related to poor overall survival of hepatocellular carcinoma. However, the involvement of the exportins in pancreatic cancer remains unknown, except for XPO1 22. Here, we analyzed the mRNA expression levels of these exportins in databases and finally identified CSE1L as our research object.

In the present study, the association between a negative prognosis and high CSE1L expression in pancreatic cancer was demonstrated. These results revealed that the CSE1L expression level was related to the outcome of patients with pancreatic cancer and may have a negative influence on pancreatic cancer patients. Therefore, CSE1L can serve as a potential prognostic indicator and treatment for pancreatic cancer.

To investigate the function of CSE1L in pancreatic cancer, we overexpressed and suppressed its expression in pancreatic cancer cells. Our experimental data revealed that knockdown of CSE1L inhibited cell proliferation and promoted apoptosis, while overexpression of CSE1L promoted cell proliferation. In addition, apoptosis assays revealed that CSE1L inhibited cell apoptosis. Wang et al confirmed that AKT/mTOR signaling is involved in CSE1L-mediated oral cancer growth 12. Therefore, we carried out western blotting assays to verify alterations of the phosphorylation levels of AKT and mTOR in PANC1, AsPC1 and CFPAC1 cells. The results revealed that the AKT/mTOR signaling pathway is crucial for CSE1L-mediated pancreatic cancer proliferation.

In conclusion, the results of the present study allowed for a better understanding of the underlying mechanism of pancreatic cancer progression. CSE1L was found to be associated with a negative prognosis in pancreatic cancer patients. In addition, CSE1L might promoted proliferation of pancreatic cancer by targeting the AKT signaling pathway. More importantly, these findings suggest that CSE1L may serve as a potential prognostic and therapeutic target for pancreatic cancer.

## Supplementary Material

Supplementary table S1.

## Figures and Tables

**Table 1:** Correlation between the clinicopathological characteristics and CSE1L expression level in pancreatic cancer.

Characteristic	Case n	CSE1L expression level		χ2 value	P value
		Low	High		
Age/year					
< 60	29	14	15	1.221	0.269
≥ 60	61	22	39		
Gender					
Male	62	22	40	1.694	0.193
Female	28	14	14		
Clinical stage					
Early stages (≤I)	10	8	2	7.5	0.006*
Advanced stages (>I)	80	28	52		
Invasion depth					
T1+T2	17	12	5	8.171	0.004*
T3+T4	73	24	49		
Lymph nodes metastasis					
Yes	53	20	33	0.275	0.6
No	37	16	21		
Distant metastasis					
Yes	3	2	1	1.2	0.338
No	87	34	53		
Differentiation					
Well	32	12	20	8.354	0.015*
Moderately	50	17	33		
Poorly	8	7	1		
