# Supplementary material for: CSE1L, as a novel prognostic marker, promotes pancreatic cancer proliferation by regulating the AKT/mTOR signaling pathway
Source: J Cancer. 2021 Mar 14;12(10):2797–806. doi: 10.7150/jca.54482 (PMC8040880; doi:10.7150/jca.54482)
Supplement: Supplementary file 1 — Supplementary table S1. [file jcav12p2797s1.pdf]

Supplementary Table 1 The primers used in this study.

	Forward-primer	Reverse-primer
CSE1L	GCAGCTCATGCTCTTGAACG	GCCAGGAAGTGTGAGAGCTT
β-actin	GTCATTCCAAATATGAGATGCGT	ATCGGAGCGTAGGATGGAAT

Supplementary Table 2 The primary antibodys used in this study.

**Name**	**Company**	**Catalog Number**	**Assay**
CSE1L	Abcam	Ab70547	WB,
CSE1L	proteintech	22219-1-AP	WB, IHC
AKT	Cell Signaling Technology	9272S	WB
P-AKT(Ser473)	Cell Signaling Technology	4060S	WB
mTOR	Cell Signaling Technology	2983T	WB
P- mTOR	Cell Signaling Technology	5536T	WB
β-actin	Sigma-aldrich	#A3854	WB
